# The long and winding road to the mitochondrial pyruvate carrier

**DOI:** 10.1186/2049-3002-1-6

**Published:** 2013-01-23

**Authors:** John C Schell, Jared Rutter

**Affiliations:** 1 Department of Biochemistry, University of Utah School of Medicine, Salt Lake City, UT, USA

## Abstract

The extraction of energy and biosynthetic building blocks from fuel metabolism is a fundamental requisite for life. Through the action of cellular enzymes, complex carbon structures are broken down in reactions coupled to the production of high-energy phosphates as in ATP and GTP as well as electron carriers such as NADH and FADH_2_. These processes traverse across compartments inside the cell in order to access specific enzymes and environments. Pyruvate is the end product of cytosolic glycolysis and has a variety of possible fates, the major one being mitochondrial oxidation. While this metabolite has been known to cross the inner mitochondrial membrane for decades, it is only recently that proteins necessary for this activity have been identified. This review will chronicle more than 40 years of research interrogating this critical process and will discuss some of the possible implications of this discovery for cancer metabolism.

## Review

An extensive body of work has accumulated over decades on the subject of mitochondrial pyruvate transport. Since the earliest proposition of a specific transporter for pyruvate in 1971 there has been continuous debate about its existence and nature [1]. Updates to techniques measuring pyruvate transport have led to revisions regarding kinetics, the metabolites that modulate transport activity, and the molecular sizes of the proposed transporter culminating in the identification of a putative transporter [2]. Unfortunately, this member of the mitochondrial carrier family (MCF) was later demonstrated to be a NAD^+^ transporter, whose activity resembled the pyruvate transporter in *in vitro* assays due an effect on the NAD^+^ dependent pyruvate dehydrogenase complex [3]. At the same time clinicians studying metabolic diseases were frustrated by mysterious cases of defective pyruvate metabolism, which displayed normal pyruvate dehydrogenase (PDH) enzyme activity and did not have mutations in any known components of the pyruvate metabolic system [4]. The obvious remaining candidate, the mitochondrial pyruvate carrier, could not be sequenced for mutations because the gene or genes encoding it had not been identified. In this review, we will take a historical perspective to describe the fits and starts that recently culminated in the recent identification of the long-sought mitochondrial pyruvate carrier (MPC) [5,6].

### Membrane transport

Membranes provide the cell with the essential ability to delineate the unregulated external environment from the specific and homeostatically controlled internal milieu. Within the cell, compartments can be further subdivided and therefore assigned specialized functions. This separation is essential for generating and utilizing electrical potential via regulated ion current, protection of precious replicative information from mutagenic insults, enforcing colocalization of molecules, and conversion of high energy electrons into high energy phosphates using proton flow. The benefits of separable intracellular compartments are only truly achieved when the transport of molecules across membranes is regulated. This regulation occurs by a variety of mechanisms, including but not limited to: post-translational modifications, increased mRNA and protein synthesis, altering transporter stability, and deploying transporters stored in vesicles. Of particular relevance for the present subject, the regulation of metabolite movement and subsequent access to enzymes is a powerful and commonly employed method for biological regulation. While we often focus on the enzymes that act on metabolites, we must not take for granted the fact that they must first be given access to these enzymes.

Membrane transport is a complex process with a somewhat confusing nomenclature. The term ‘transporter’ is commonly used to describe most proteins that facilitate movement across a membrane but this can further be segregated into carriers and channels. Channels are used to conduct ions and cycle between open and closed states, with some also exhibiting an inactivation step. They form a completely continuous tunnel through a bilayer that allows for rapid conductance of many ions. A carrier, in contrast, does not form a complete pore but is open to one side of the bilayer at a time and substances are transported during the cycling of these conformations. Channels are typically found in circumstances where large amounts of ions must be rapidly conducted as in electrical signaling while carriers are often utilized in situations wherein capacity is less critical [7].

### Pyruvate metabolism

**Figure 1:**
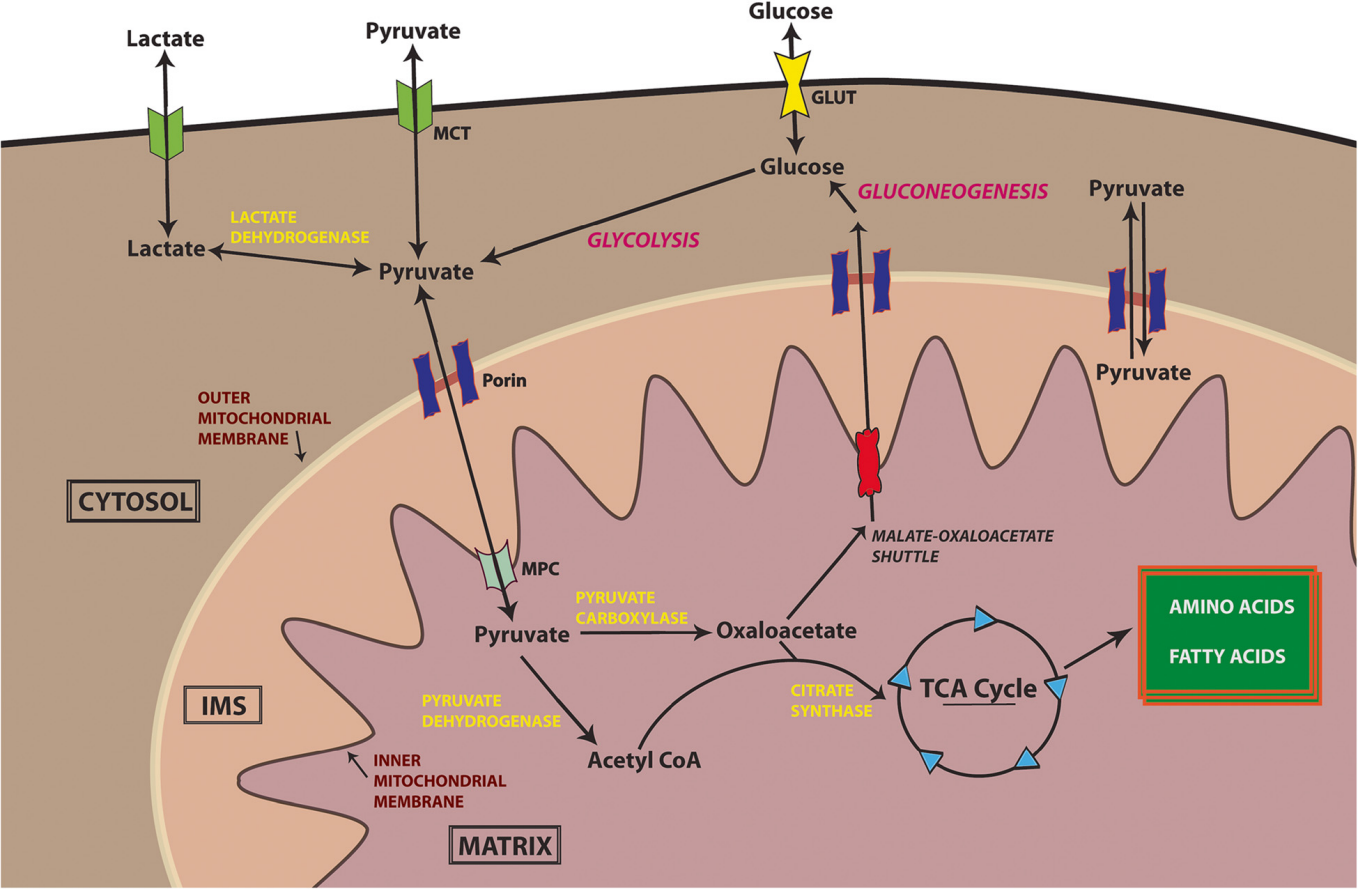
Overview of cellular pyruvate movement and metabolism.

Pyruvate is a key node in the branching pathways of glucose, fatty acid and amino acid metabolism. The overall metabolic profile of the cell dictates its metabolism, which is highly dynamic to allow this molecule to be generated and used when and where it is most needed. Perhaps the most straightforward and quantitatively important source of pyruvate is from the breakdown of carbohydrates such as glucose through glycolysis. Glucose enters the cytosol through specific transporters (the GLUT family) and is processed by one of several pathways depending on cellular requirements. Glycolysis occurs in the cytosol and produces a limited amount of ATP, but the end product is two 3-carbon molecules of pyruvate, which can be diverted yet again into many pathways depending on the requirements of the cell. In aerobic conditions, pyruvate is primarily transported into the mitochondrial matrix and converted to acetyl-coenzyme A (acetyl-CoA) and carbon dioxide by the pyruvate dehydrogenase complex (PDC). For access to the mitochondrial matrix, three layers must be traversed: the outer mitochondrial membrane, the intermembrane space, and the inner mitochondrial membrane (Figure 1). Most of the metabolic activities of mitochondria occur in the matrix, either by soluble proteins or enzymes embedded in the inner membrane.

Transport of pyruvate across the outer mitochondrial membrane appears to be easily accomplished via large non-selective channels such as voltage-dependent anion channels/porin, which enable passive diffusion [8]. Indeed, deficiencies in these channels have been suggested to block pyruvate metabolism [9]. The movement through the inner mitochondrial membrane is far more restrictive, however. The tight control over matrix accessibility makes intuitive sense, as the matrix is the site wherein the majority of mitochondrial metabolic enzymes are localized. Many metabolites have specific mitochondrial inner membrane transporters that have been identified and well studied [10].

Once within the mitochondrial matrix, pyruvate may be diverted into two disparate paths. On one path pyruvate is carboxylated to form oxaloacetate, which can be used for gluconeogenesis. On the other path, pyruvate is decarboxylated to form Acetyl-CoA. After conversion to acetyl-CoA in the mitochondrial matrix, pyruvate’s potential fates are constrained. While components of the tricarboxylic acid (TCA) cycle may be used to produce amino acids, fatty acids, or glucose, the carbons from pyruvate are primarily converted to carbon dioxide. In the process, the energy from pyruvate is converted into high energy reducing intermediates that power the electron transport chain. Due to the impact of this decision, the choice to convert pyruvate into acetyl-CoA is tightly regulated [11].

In oxygen-depleted environments, cytosolic pyruvate is converted into lactate and exported from the cell in a process known as anaerobic glycolysis. Without the requisite oxygen necessary to oxidize the pyruvate carbons, the cell is required to generate energy via this method. Certain cell types also engage in a form of glucose metabolism known as aerobic glycolysis in which glycolysis-derived pyruvate is converted to lactate even when oxygen is adequate for pyruvate oxidation. This seemingly wasteful decision is a common component of the metabolic alterations in a variety of cancers. No discussion of cancer metabolism, especially one focusing on pyruvate, would be complete without a reference to Otto Warburg and his observation that tumor cells utilize aerobic glycolysis, subsequently named the ‘Warburg effect’ [12]. Currently there is intense interest in understanding what role the Warburg effect plays in cancer initiation and growth, with therapeutics being developed in the hope of reversing metabolic changes required by the cancer cells [13,14]. This work is enabled by our rather sophisticated understanding of many aspects of central carbon metabolism and its regulation. One question of fundamental importance has remained, however: how does pyruvate move across the inner mitochondrial membrane?

### The problem

In diagrams of metabolism, the canonical depiction of pyruvate transport into the mitochondria is a solid arrow traversing the mitochondrial inner membrane. Initially it was proposed that pyruvate was able to cross the membrane in its undissociated (acid) form but evaluation of its biochemical properties show that it is largely in its ionic form within the cell and should therefore require a transporter [15,16]. While a minor detail to most, the solid arrows traversing membranes are a source of frustration to those that seek to understand how substrate partitioning can influence metabolic decisions. Although much remains unknown, two independent studies have rigorously tested two mitochondrial inner membrane proteins [5,6]. Originally named BRP44L and BRP44 (now named MPC1 and MPC2, respectively), these proteins have been shown to form a complex that is necessary and sufficient for the movement of pyruvate across the mitochondrial inner membrane and into the matrix.

Decades of research have investigated the mitochondrial pyruvate carrier (MPC), all without knowing the encoding gene or genes. These studies range from observational inquiries in general or specific situations to intensive and focused studies on the kinetics and biochemistry of the MPC. In spite of the challenge of not knowing the genes or structure, these pioneering scientists generated an extensive base of knowledge regarding the mitochondrial pyruvate carrier and its functional characteristics. Due to space considerations, we are only able to comment on a few of the elegant studies conducted by a number of rigorous scientists over decades of endeavor.

## Prior studies of mitochondrial pyruvate transport

### Diffusion across the membrane or transport?

Like many fields of study, the mitochondrial pyruvate carrier has been the subject of significant disagreement over the last 40 years. In spite of the controversies, these studies have enhanced the understanding of the MPC and have honed the techniques for its analysis. The earliest assumption of pyruvate movement was that it could traverse the inner membrane in its undissociated form despite the acknowledgement that the inner membrane was a selective barrier for the movement of other metabolites [15]. Using protein-free liposomes it was found that pyruvate did indeed have the ability to cross membranes directly [17]. A potential caveat of these studies was the use of 100 mM pyruvate, a concentration that is roughly 1,000-fold higher than would is typically encountered within the cell [18,19]. In addition, artificial membranes used in these studies may not replicate the permeability and other properties of the mitochondrial inner membrane. Indeed, later studies unequivocally demonstrated transporter-mediated pyruvate movement.

The interest in the mitochondria and metabolite transporters extended to pyruvate movement and in 1971 Papa *et al*. provided evidence that pyruvate moved across the mitochondrial membrane via a specific translocator and is coupled to ion exchange [1]. In this study the authors purified mitochondria from rat liver and preincubated the suspension with inhibitors to block pyruvate metabolism and inhibit the electron transport chain and subsequent ATP production. Radiolabeled pyruvate was then added to the mitochondria and the mixture was centrifuged either directly or through a silicone oil differential gradient into HClO_4 _to control the kinetics of the reaction and stop transport abruptly. This initial study showed pyruvate translocation and exchange displayed saturation kinetics, which they argued established the existence of a specific pyruvate transporter. The data provided by this work was also the first suggestion that pyruvate movement across the inner mitochondrial membrane was coupled to a proton gradient. This coupling to the proton gradient was rationalized as a positive feedback loop to supply active, energetically coupled and respiring mitochondria with additional fuel.

This initial suggestion of a mitochondrial pyruvate carrier was countered by a report that pyruvate adsorption to the membrane and not transport across it was responsible for the findings [20]. The contrary conclusions were partially based on the finding that boiled and denatured mitochondria accumulate pyruvate to a similar level as active mitochondria in a pH-dependent manner. While this study provides an alternative mechanistic explanation for the *in vitro* observations, their findings did not preclude the existence of a carrier. Nevertheless, this paper presented a challenge that would need to be answered in order to justify continued study of the putative MPC.

### Identification of specific inhibitors

Shortly after this report a key advance was made that solidified the argument for a specific transporter. Halestrap and colleagues identified and characterized an inhibitor of mitochondrial pyruvate transport. α-Cyano-4-hydroxycinnamate (CHC) was observed to inhibit pyruvate oxidation and found to do this by blocking mitochondrial pyruvate transport. In whole mitochondria, the inhibitor blocks transport and therefore metabolism but the inhibitor has no effect on ruptured mitochondria [21]. In this way it is possible to ascribe the inhibitory effect to be at the membrane rather than a mitochondrial enzyme. CHC and other cinnamate-based inhibitors resemble the enol form of pyruvate with an attached aromatic ring [22]. In the absence of reverse genetics, chemical modulation was the only available methodology to perturb protein function and measure the effects. The lack of an inhibitor had been a major basis for the earlier claim that a specific mitochondrial pyruvate carrier did not exist [15]. With an inhibitor, the properties of the carrier could be investigated more rigorously and separated from diffusion through the membrane, adsorption or the activity of other transporters.

### Inhibitor-stop technique and modulation by other metabolites

The discovery of an inhibitor enabled the use of the ‘inhibitor-stop’ technique to carry out detailed biochemical studies on the carrier [23]. This technique had been used to characterize the transport of other metabolites and allows metabolite exchange for a specified time followed by introduction of the inhibitor to stop the transport reaction and rapid sedimentation of the mitochondria. Beyond this specific inhibitor, the effect of other metabolites on pyruvate uptake and oxidation was investigated. Almost simultaneous studies showed the inhibitory effects on pyruvate transport of α-ketoacids and phenylpyruvate, lending additional physiological validity to the presence of a transporter [24,25]. These observations relate to the first implication of the mitochondrial pyruvate carrier in human disease. Specifically, toxic accumulation of phenylpyruvate in phenylketonuria patients could disrupt oxidative metabolism by preventing normal pyruvate movement into the mitochondria [25,26]. Malate, an intermediate in the citric acid cycle, was shown to significantly increase mitochondrial uptake of pyruvate while not affecting affinity. The initial characterization of the kinetics of pyruvate transport showed that it appeared to follow first-order kinetics and have a higher rate constant than mitochondrial succinate or citrate transport [24]. As the rate of transport across a membrane is notoriously difficult to determine, later studies would revise these values.

Expanding upon the discovery of cinnamates as MPC inhibitors, it was shown that these compounds also inhibit the transport of pyruvate across the plasma membrane of erythrocytes, but they do so much less potently, suggesting that the two membranes have different transporters [27]. This study also implicated the MPC in gluconeogenesis. Because pyruvate must be converted to oxaloacetate in the mitochondria and exported to the cytosol for conversion to glucose, inhibition of gluconeogenesis is completely expected. Utilizing the knowledge that the plasma membrane pyruvate transporter in erythrocytes is less sensitive to the inhibitors it was possible to use a concentration that would only prevent mitochondrial transport and show that this lower concentration had a similar effect on gluconeogenesis, once again implicating the mitochondria as a mediator of this essential process [21].

Using an altered definition for pyruvate transport that was limited to what could be blocked by the inhibitor, Pande and Parvin provided new data for the inhibition of the transporter by cinnamates [28]. Even after CHC-treated mitochondria were washed with 60 mM pyruvate, concentrations of pyruvate that would normally drive pyruvate oxidation (1 mM) failed to do so, suggesting the mechanism of inhibition is non-competitive. The same study also showed that respiration experiments in the presence of 60 mM pyruvate became insensitive to CHC. Therefore, while CHC doesn’t appear to be a competitive inhibitor, high concentrations of pyruvate (60 mM) can overcome inhibition of transport. These authors attributed this phenomenon to a carrier-independent and inhibitor-insensitive method of pyruvate movement into the mitochondria. Distinction of carrier-dependent and independent pyruvate movement is a notable advance necessary for developing experimental systems to investigate pyruvate transport and to identify the involved factors. This property also represents a key physiological basis for the necessity of a specific transporter. At the low physiological concentrations of pyruvate found within the cytosol, diffusion would be unable to adequately transport this essential metabolite to its site of metabolism in the matrix.

### Pyruvate transport as a rate-limiting step in pyruvate oxidation

Further studies of the kinetics of pyruvate uptake found the rate to be quite low (V_max_ = 0.54 ± 0.03 nmol per minute per mg of mitochondrial protein) compared to other transport systems [29]. By analogy with glutamate transport, which is also rather sluggish and thought to be the rate-limiting step in glutamine metabolism, this raised the possibility that mitochondrial pyruvate transport might be rate limiting for pathways requiring mitochondrial pyruvate, like oxidation and gluconeogenesis [30]. Data in favor of rate-limiting control for the MPC later came from Pande and Parvin [28]. Using the revised method for determining pyruvate uptake described above, they determined even slower rates for MPC activity. This technique and accompanying paper questioned much of the previous work on the transporter. The rate limitation of pyruvate uptake on oxygen consumption in the presence of ADP was later confirmed [31].

At this point, many important questions regarding the properties of the pyruvate transporter remained problematic. Various studies were providing contradictory conclusions regarding metabolite effects on pyruvate transport [25,28,29,32]. Providing definitive answers surrounding the phenomenon of mitochondrial pyruvate transport would require the identity of the genes and proteins involved to allow biochemical purification and reconstitution in an isolated system. Studying transport properties in the context of the mitochondrial membrane is extremely difficult due to the presence of other transporters and ongoing metabolism. The purification, identification and reconstitution of the transporter would solve many of these issues. As a result, great effort was expended to accomplish these goals.

### Purification

Purification of the transporter and reconstitution into liposomes would provide the single best system for characterizing transport properties as well as sharpening the focus onto this one activity. Such an isolated system is perhaps the highest threshold to be achieved in biochemical studies and is one of the most difficult enterprises to undertake, made even more difficult by the lack of identity of the proteins. Without knowing their identity, overexpressing and tagging of the proteins for purification would not be possible. In spite of the challenges, the purification and reconstitution of pyruvate transport activity from mitochondria was demonstrated in 1986 [33,34]. Specifically, pyruvate exchange activity across proteoliposomal membranes was studied and shown to be sensitive to 2-cyano-4-hydroxycinnamate. While this represented a critical step in the studies of mitochondrial pyruvate transport, the MPC was not purified. Attempts to reconstitute a purified preparation of the MPC were first described in castor bean mitochondria [34]. When subjected to SDS-PAGE, the active, partially purified mixture contained proteins of 74, 66, 34, 32, 30 and 12 kDa. These data led the authors to conclude that one or more of the proteins was the pyruvate transporter, but complete purification remained elusive. New strategies would likely be required to achieve this goal.

Two papers in 1984 and 1986 described the binding properties of the MPC for α-cyanocinnamate [35,36]. Studies using a ^14^C labeled inhibitor provided information on binding and dissociation beyond what could be learned from activity measurements. Using this system, the authors drew several parallels between inhibitor binding and inhibition of pyruvate transport, thus providing support for a direct relationship between the inhibitor, the MPC and pyruvate transport [35]. Additionally, preincubation of mitochondria with UK5099 for 30 minutes caused stable MPC inhibition that was not reversed by subsequent 100-fold dilution (while control experiments showed that NADH and succinate metabolism was preserved) [34]. The data supporting a stable inhibitor-bound complex led to the idea of using an immobilized inhibitor as a means of purifying the MPC.

The Azzi group that had previously reconstituted pyruvate transport activity using hydoxyapatite chromatography refined their methods in an attempt to identify the specific proteins involved. For these studies they covalently immobilized the inhibitor 2-cyano-4-hydroxycinnamate on sepharose. Following one-step hydroxyapatite purification, the mitochondrial fraction was passed through the cinnamate column. Both 34 kDa and 31.5 kDa proteins were observed following elution [37]. We currently have no way to explain the molecular weights of the proteins found in this study in light of the smaller size of the newly discovered MPC proteins, but it is possible that the observed proteins were monocarboxylate (MCT) transporters, which are predominantly at the plasma membrane, but have been proposed to exist in mitochondria [38]. The authors did find that higher concentrations of inhibitor were required to block transport activity compared to isolated intact mitochondria and Halestrap previously showed that this is the case for the MCT pyruvate transporter compared to the mitochondrial carrier. These initial studies in bovine heart were followed by similar work in rat liver with similar results showing proteins in the 29 to 37 kDa size range catalyzing pyruvate transport [39]. Now with the identification of the MPC it will be possible to go back and re-evaluate these studies. The MCTs may also represent another mode of pyruvate transport with low sensitivity that is active at higher concentrations although there is no definitive data on this. Similar studies in *Saccharomyces cerevisiae* identified two polypeptides with a molecular mass of 26 kDa and 50 kDa, failing to purify the 34 kDa protein identified as the transporter in bovine heart. In addition to the size discrepancy, it was found that their activities were different, with the yeast carrier being more active but less abundant than what was purified and reconstituted from bovine and rat tissue [40].

### Fits and starts toward identification

α-Cyanocinnamate was shown to protect the pyruvate transporter from the thiol-blocking reagent *N*-phenylmaleimide and diminished labeling of a 15 kDa protein by radiolabeled *N*-phenylmaleimide in rat liver and heart mitochondria. This experiment also revealed a 12 kDa protein in heart mitochondria that was not detected in liver leading the authors to conclude it was most likely a proteolytic breakdown product [41]. Unfortunately, the same authors later found that the 15 kDa protein was a subunit of cytochrome oxidase (COXIV) [2].

Even with these purification procedures in hand, the identity of the transporter remained a mystery. From what had been uncovered regarding mitochondrial transport of other metabolites, it seemed likely that pyruvate transport would be facilitated by a member of the mitochondrial carrier family (MCF) [42]. In 2003, a report was published claiming to have finally identified the mitochondrial pyruvate carrier [2]. Using *S. cerevesiae*, the authors focused specifically on the MCF protein family, comprised of 35 proteins in yeast. In total, 16 had been previously characterized and had known substrates. Of the remaining proteins, 1 was essential for growth under respiratory conditions, which left 18 to characterize. A systematic analysis of strains individually lacking each of the 18 MCF proteins produced 1 candidate, the 41.9 kDa protein encoded by YIL006w. This mutant displayed the lowest level of pyruvate uptake and weakest response to UK5099, but had limited conservation from yeast to *Drosophila*, mouse, and man. Unfortunately, 3 years later it was found that this protein likely functions to transport NAD^+^ into the mitochondrial matrix, which was definitively proven by liposomal reconstitution [3]. Indeed, phylogenetic analysis showed a clustering of this MCF protein with other nucleotide transporters. The alterations in ‘pyruvate transport’ originally observed were likely a result of reduced activity of the NAD^+^-requiring pyruvate dehydrogenase complex. This technique was used to characterize other MCF proteins but failed to identify the MPC [43].

The approach employed by Hilyard *et al*. highlights the potential pitfalls and difficulty of searching for protein functions using family characteristics and sequence homology. It cannot, however, be understated how difficult these transporter studies were, especially with unidentified proteins. Characterizing basic biochemical attributes of membrane proteins is difficult and tedious work and achieving reproducible results requires zealous oversight. The work done over several decades by Halestrap, Palmieri, Papa, Azzi and their colleagues and many others represents an incredible body of work that facilitated the identification of the MPC.

## Identification of the mitochondrial pyruvate carrier

Following these two papers, one claiming to have identified the mitochondrial pyruvate carrier and the second calling this into question, studies on the carrier slowed. Those that were carried out became increasingly focused: pyruvate transport in frog mitochondria and cinnamate-resistant sake yeast [44,45]. The eventual identification of the MPC came from two groups, neither one of which had set out to identify the carrier. Bricker *et al*. began with highly conserved mitochondrial proteins with the logic that sequence conservation was an indicator of essential function [5]. Genetic ablation of the MPC genes in yeast and flies led to an increase in glycolytic intermediates and pyruvate with reductions in acetyl-CoA and TCA cycle metabolites. Herzig *et al*. came upon the MPC by following a defect in the synthesis in lipoic acid and growth defects in the absence of valine and leucine [6]. These two different approaches and very different experimental strategies led to the same conclusion: the proteins under investigation assemble to form the elusive MPC.

The proteins found to be required for pyruvate transport are quite small. Bricker *et al*. showed the proteins were roughly 15 kDa by SDS PAGE and interacted to form a larger complex consistent with some of the earlier studies conducted by Brailsford *et al*. and Halestrap *et al*. [34,41]. The MPC inhibitor UK5099 was used differently by the two groups, but the data generated was complementary in establishing the necessity and sufficiency of the MPC proteins for mitochondrial pyruvate uptake. Bricker *et al*. used yeast genetics to identify a mutation of *MPC1* that conferred UK5099 resistance for growth and *in vitro* mitochondrial pyruvate uptake. Herzig *et al*. established the sufficiency of the MPC for mitochondrial pyruvate uptake. They expressed murine MPC1 and MPC2 in *Lactococcus lactis* and showed that the two genes could confer pyruvate uptake, but neither gene alone had any effect. They also showed that this reconstituted MPC was sensitive to UK5099.

Bricker *et al*. also identified two point mutations in human *MPC1* that cause a metabolic syndrome that is highly reminiscent of known defects in pyruvate metabolism [4]. Sequencing revealed two mutations in highly conserved regions of *MPC1* and metabolic studies showed that the defect in pyruvate metabolism could be rescued by expression of wild-type *MPC1*. These data combine to provide compelling evidence that the MPC1/MPC2 complex is the major mitochondrial pyruvate transporter [46].

### Implications of the discovery of the MPC genes

Despite the hidden identity of the mitochondrial pyruvate carrier, many studies have provided important information about the physiological significance and regulation of this process. These studies touch on the relationship with gluconeogenesis, MPC control by hormones and drugs and include relevance to pathological conditions including hyperthyroidism, aging, and diabetes [47-55]. The clinical relationship has also been investigated with respect to inborn errors of metabolism. As expected, a defect in mitochondrial pyruvate transport causes a phenotype that is similar to a mutation in one of the components of the pyruvate dehydrogenase complex [4,5]. Now that the MPC genes have been identified, we are now in a position to determine whether patients that appear to have a PDH mutation but retain normal enzymatic activity have a mutation in one of these genes. As the epidemiology of PDH mutations remains unknown it is difficult to estimate how many potential idiopathic pyruvate metabolism defects with normal PDH activity, of which there are many, may be due to mutations of MPC [56].

Apart from basic metabolic disorders, the MPC may also exert a significant pathophysiological effect on the metabolic alterations found in cancer. At the most simplistic level, the Warburg effect could be described as a loss or decrease of MPC function. Many other well-written reviews have discussed cancer metabolism in depth so we will attempt to focus on aspects with relevant to mitochondrial pyruvate transport [57,58]. The study of metabolism in cancer has expanded significantly with many studies examining the role of glycolysis, oxidative phosphorylation, fatty acid oxidation, the TCA cycle, and hypoxia but previous studies have not been able to provide models that integrate mitochondrial pyruvate transport [59-61].

The limited work on mitochondrial pyruvate transport in cancer has supported the expectation that changes in the MPC may promote the glycolytic metabolic profile. One early study found that the activity of the mitochondrial pyruvate transporter was an order of magnitude lower in Ehrlich tumor cells compared to normal liver cells implicating the MPC in the metabolic phenotype of these cells [62]. A follow-up study comparing Ehrlich ascites, Morris hepatoma 44, and Morris hepatoma 3924A cells with normal rat liver cells found that the V_max _of the transporter was decreased and pyruvate supported respiration was similarly reduced in each of the cancer cell preparations. There were no significant changes in transmembrane pH gradient, which may have explained reduced transport activity. The authors concluded the defect was due to lower transporter activity due to either a reduction in the abundance of the carrier or due to alterations in the cellular environment that may affect the transporter [63]. In both the Morris hepatoma 44 and Morris hepatoma 3924A cells, the K_M _for pyruvate transport was increased, implying that the MPC might actually be different in those cells and exhibit a lower affinity for pyruvate.

Despite the limited data available on the connection between mitochondrial pyruvate transport and cancer metabolism, the possible relationships and their implications are exciting. The proximity of the MPC to metabolic enzymes with a validated role in cancer metabolism draws immediate attention to the MPC and its modulation in cancer. Lactate dehydrogenase, the M2 isoform of pyruvate kinase, and pyruvate dehydrogenase act directly on pyruvate metabolism and could indirectly affect its movement into mitochondria [64-66]. Each of these enzymes is altered in cancers, thereby perturbing pyruvate metabolism. We therefore consider it reasonable to anticipate a role for the MPC in cancer-relevant control of pyruvate metabolism. Many outstanding issues regarding how the MPC may contribute to the Warburg effect remain to be addressed, including how these various enzymes and their regulation interact in the context of cancer metabolism. For example, reduced MPC expression or activity in the face of altered cytosolic pyruvate metabolism, namely through increased pyruvate kinase isozyme M2 (PKM2) and lactate dehydrogenase (LDH), might provide only a marginal exacerbation of the Warburg effect. In contrast, reducing MPC in cancers with preserved cytosolic pyruvate metabolism might profoundly increase lactate production and the manifestation of the Warburg effect. There are many avenues of investigation available now with the identification of proteins necessary for the mitochondrial import of pyruvate. Understanding how the MPC fits into the complex environment of cancer metabolism sits at the forefront.

## Conclusions

The metabolic profile of cancer has recently come back into vogue with people espousing the century-old contributions of biochemists, most notably Otto Warburg. Many sophisticated studies in the past few years have placed cancer metabolism in a deserved position of prominence in the field of oncology. The initial observation that tumor cells produce high levels of lactate despite adequate oxygenation was essential for future investigations of the mechanisms and physiological importance of the Warburg effect. Key tumor suppressors have been shown to regulate metabolism and by doing so alter the fate of the cell. By controlling the mitochondrial flow of pyruvate, a cancer cell can tune its biology to meet the demands of rapid growth. Now with the identification of the mitochondrial pyruvate carrier we are poised to add yet another integral piece to this story and in doing so hopefully gain a better understanding that will ultimately translate into therapy.

## Competing interests

The author(s) declare that they have no competing interests.

## Authors’ contributions

JCS and JR wrote and edited the manuscript. Both authors read and approved the final manuscript.
